# Ethnomedicinal uses, phytochemistry, and pharmacology of the genus *Sarcophyte*: a review 

**DOI:** 10.3389/fphar.2023.1301672

**Published:** 2024-01-08

**Authors:** Onyancha Jared Misonge, Moriasi Gervason Apiri, Meroka James Onsinyo, Wainaina Samuel Murigi, Sengera Geoffrey Ogeto, Nyandoro Vincent Obaga

**Affiliations:** ^1^ Department of Pharmacognosy, School of Pharmacy, Mount Kenya University, Thika, Kenya; ^2^ Department of Medical Biochemistry, Mount Kenya University, Thika, Kenya; ^3^ Department of Biochemistry, Microbiology, and Biotechnology, Kenyatta University, Nairobi, Kenya; ^4^ School of Pharmacy, Kabarak University, Nakuru, Kenya; ^5^ Department of Pharmacy, Kisii University, Kisii, Kenya

**Keywords:** anticancer, anti-inflammatory, antimicrobial, diinsinin, diinsininol, holoparasite, phytochemicals

## Abstract

Although medicinal plants have been used by ethnic communities since ancient times to prevent and treat various diseases, only a few have been scientifically documented. Therefore, due to their rare availability and lack of comprehensive scientific information, we reviewed the ethnomedicinal uses, phytochemistry, and pharmacological activities of plants within the genus *Sarcophyte*. To do this, we used specific search terms and phrases to retrieve relevant information from online sources published in English from 2000 to July 2023. The results showed that there are only two plants in the genus *Sarcophyte* (*Sarcophyte sanguinea* Sparrm. and *Sarcophyte piriei* Hutch.), which are traditionally used to treat a wide range of diseases, especially cancer, and skin, gastrointestinal, and urinogenital tract ailments in humans, and to cure animals in ethnoveterinary practices. It was noted that 13 secondary metabolites have been isolated from the two plants, the most prominent of which are flavonoids (diinsininol, diinsinin, and naringenin). The antioxidant activity of *S*. *piriei* is reported based on the scavenging of 2,2-diphenyl-1-picrylhydrazyl (DPPH) (IC_50_: 4.26 ± 0.22 μg/mL) and 2 -2′-Azino-di-[3-ethylbenzthiazoline sulfonate (ABTS) radicals (IC_50_: 4.62 ± 0.14 μg/mL), chelating iron (IC_50_: 1.82 ± 0.01 μg/mL, 3.50 ± 0.09 μg/mL), and nitric oxide (IC_50_: 9.97 ± 0.88 μg/mL, 9.09 ± 0.11 μg/mL). The methanolic stem extracts of *S. piriei* possess antimicrobial activity against *Staphylococcus aureus, Escherichia coli, Klebsiella pneumoniae, Vibrio fluvialis,* and *Enterococcus avium*, with minimum inhibitory concentration (MIC) values ranging from 0.16 to 0.625 mg/mL, and a minimum bactericidal concentration (MBC) of 1.25 to 5 mg/mL. Cytotoxic effects of the extracts from the two plant species were also demonstrated. *Sarcophyte piriei* possesses therapeutic potential as evidenced by the inhibitory effects of the aqueous rhizome extract on edema (1,000 mg/kg) and prostaglandin synthesis (IC_50_ = 0.2 mg/mL). In addition, diinsininol and diinsinin were isolated from *S. sanguinea* inhibited prostaglandin synthesis (IC_50_: 9.20 µM, 13.14 µM) and platelet-activating factor-induced exocytosis. Therefore, based on this review, further scientific research is needed to demystify the links between traditional medicinal uses, various secondary metabolites, and the pharmacology of the two plants.

## 1 Background

The use of medicinal plants as therapeutic agents for human and animal diseases is an extensive and diverse phenomenon, demonstrating their beneficial role in meeting primary healthcare needs (Jamaddar et al., 2023). Moreover, medicinal plants play a significant role in the conventional healthcare system as they are a valuable source of various allopathic drugs currently used to treat diseases (Dey et al., 2021). Considering the high cost of conventional healthcare, especially in low- and middle-income countries (LIMCs), the higher efficacy in managing various diseases (Ekor, 2014; Patrício et al., 2022), and the cultural acceptability of botanical drugs, traditional medicine still plays an important role worldwide (Dey et al., 2021; Siddique et al., 2021; Ndhlovu et al., 2023).

The vast therapeutic potential of medicinal plants has attracted immense research interest from the scientific community to unearth alternative effective lead compounds for drug development, especially against devastating diseases (Abdalla and Mühling, 2019; Swamy, 2020; Salama et al., 2021). It is now well recognized that the pharmacological effects of medicinal plants are mediated by various bioactive secondary metabolites (Swamy, 2020). Consequently, research has demystified the pharmacological role of various phytochemicals in preventing, slowing down, or averting the pathogenesis of many diseases (Howes and Perry, 2011; Gaikwad et al., 2014; Howes, 2017; Ahmad et al., 2020; Moriasi et al., 2020a; Moriasi et al., 2021a). For instance, antioxidant-associated phytochemicals quench oxidative stress in the body, thereby preventing undesirable sequelae (Moriasi et al., 2020b). Amelioration of oxidative stress is key to preventing and reversing associated diseases such as diabetes mellitus, neurodegeneration, cancer, and metabolic syndrome, among others (Moriasi et al., 2020b; Moriasi et al., 2021b). Therefore, certain phytochemicals in medicinal plants can help predict the efficacy and potency of that plant against specific or a range of diseases.

The existence of traditional medicine depends primarily on the diversity of medicinal plants and the associated ethnomedicinal information on their preparation and use (Josephine Ozioma and Antoinette Nwamaka Chinwe, 2019). Thus, medicinal plants are an indispensable resource for maintaining the health and overall wellbeing of people and animals in various ethnic groups worldwide (WHO, 2013; Josephine Ozioma and Antoinette Nwamaka Chinwe, 2019; Okot et al., 2020). The popularity and high dependency on traditional medicine, especially in sub-Saharan Africa (James et al., 2018), denote its significant role in healthcare. However, rapidly increasing human populations, urbanization, climate change, and habitat destruction are harming essential medicinal plants and their resources (Okello and Kiringe, 2004; Kamau et al., 2016; Razgour et al., 2020). To address this, it is imperative to carefully investigate and document the ethnomedicinal information, pharmacological activities, and phytochemistry of promising plants to preserve the knowledge and facilitate further research aimed at valorizing their efficacy as potential sources of new drugs and to support conservation programs (Kamau et al., 2016; Kigen et al., 2014; Haque et al., 2022; Hmidouche et al., 2023). Accordingly, we reviewed the local and traditional medicinal uses, pharmacological activities, and phytochemistry of plants within the genus *Sarcophyte* because of the lack of comprehensive scientific information and because they are rarely encountered.

The Sarcophyte genus belongs to the plant family Balanophoraceae and the order Santalales (sandalwood), according to the Angiosperm Phylogeny Group IV classification system (Angiosperm Phylogeny Group, 2016; Nickrent, 2020). The family comprises 18 genera with over 100 species of root holoparasitic geophytes with a pantropical distribution (Dennis et al., 2023). The genus *Sarcophyte* comprises a single species of two plants: *Sarcophyte sanguinea* Sparrm.) and *Sarcophyte piriei* (Hutch.). The two plants are native to eastern and southern Africa, but they are rarely encountered due to their patchy distribution, leading to inadequate sampling, poor preservation, and insufficient research (Maroyi, 2017).

The two plants are used in traditional medicine to treat cancer, snake bites, and disorders of the respiratory, gastrointestinal, integumentary, reproductive, and nervous systems (Ogundaini et al., 1996; Muriuki, 2011; De Wet et al., 2013; Naidoo et al., 2013; Maroyi, 2017). However, there is a paucity of comprehensive scientific literature on the ethnomedicinal uses, phytochemistry, and pharmacological activities of plants of the genus *Sarcophyte*. Thus, sufficient scientific reports on the genus *Sarcophyte* are needed to help appraise its ethnomedicinal, pharmacological, and phytochemical value.

Therefore, this review provides a comprehensive account of the plants’ ethnomedicinal uses, phytochemistry, and pharmacological activities of the two plants of the genus *Sarcophyte.* This review further highlights the existing research gaps and potential scientific opportunities that may significantly contribute to the valorization of the investigated plants as sources of alternative therapeutic agents for the treatment of various diseases.

## 2 Methodology

We retrieved the relevant literature on the ethnobotanical uses, phytochemistry, and pharmacological activities of the genus *Sarcophyte* published from 2000 to July 2023 from Google Scholar, Wiley Online Library, Web of Science, PubMed, SCOPUS, SpringerLink, SciFinder, and Science Direct, using specific search terms and phrases such as “*Sarcophyte,*” “Pharmacologic activity of *Sarcophyte,*” *“*Taxonomy of *Sarcophyte,” “*distribution/diversity of *Sarcophyte*,” “phytochemistry of *Sarcophyte,*” and “ethnomedicinal uses of *Sarcophyte*.” Additionally, an extensive search and analysis of traditional medicinal uses, phytochemistry, and pharmacological activities of plant species belonging to the genus *Sarcophyte* was performed using published articles, journals, Ph.D. and MSc. dissertations, conference papers, available data from herbaria, and books published in English. The materials were carefully screened, and only complete and relevant information was further evaluated and included in this review. Information on the worldwide distribution of plant species in the genus *Sarcophyte* was obtained from online databases, including JSTOR Global Plants (JGP) (JSTOR, 2022a), World Flora Online (WFO) (World Flora Database, 2022), Prelude Medicinal Plants (PMP) (Africamuseum, 2022), African Plant Database (APD) (APD, 2022), and Global Biodiversity Information Facility (GBIF) (GBIF, 2022). Species details recorded included collector, accepted name, species number, occurrence, and the herbarium. Species names were confirmed using the International Plant Name Index (IPNI) (IPNI, 2022) and the taxonomic data in the WFO database (IPNI, 2022). The PubChem database was used to verify the IUPAC names of secondary metabolites isolated from plants of the genus *Sarcophyte*, and their chemical structures were drawn using ChemBio Draw Ultra, version 14.0.

## 3 Botanical description

Plants of the *Sarcophyte* genus are characterized by their unique botanical features, as currently described in the World Flora Online (WFO) database (World Flora Online, 2022). Plants of this genus are perennial polyparasitic herbs that grow up to 40 cm long and attach themselves to the roots of various host plants, especially those of the Mimosaceae family. They form a large, warty, irregularly lobed tuber measuring 5–15 cm x 5–11 cm, and short stems surrounded by three to four lobed sheaths, 8–25 cm long. Some of the host plants for *Sarcophyte* include *Acacia*, *Hyphaene* spp., *Commiphora* spp., *Ficus* spp., *Faidherbia albida* (Delile) A. Chev., and *Mimusops obtusifolia* Lam.

Moreover, plants of the genus *Sarcophyte* possess numerous spirally arranged, scale-like, ovate-lanceolate leaves up to 3.5 cm long. Their inflorescence is fleshy, with a colored to bright red panicle measuring 5–10 cm in diameter, 2–6 cm long branches, and subtended by a bract. The bracts are ovate-lanceolate, measuring 0.8–2 cm x 0.5–1.2 cm, and are only scaly at the base. The male inflorescence has many secondary flowering branches in groups of two to three, unisexual, usually regular, and 3 (-4)- merous. The pedicel is short, and the perianth segments are elliptic to oblong, measuring 1.5–3.5 mm x 1.5–2 m, with a blunt to acute apex, and flesh stamens measuring 1.5–2.5 mm. The female inflorescence has many secondary branches, each with 5–12 almost globular, spadix-like clusters, 0.4–1.4 cm in diameter, with up to 200 flowers completely sunk in a common receptacle. They do not have a perianth, and their ovary is inferior and 3-celled, while the stigma is disc-shaped, the style reduced, and one-seeded. The fruits are pseudo-berry, aggregated in a rounded and reddish infructescence, with a fleshy epicarp, a hard endocarp, and crowded carpels of the separate flowers that are not consolidated as in a compound fruit (Schmelzer and Gurib-Fakim, 2013). The stamens of *S*. *sanguinea* are more than half of the perianth segments, and the flowers have a stinky, unpleasant odor, whereas the stamens of *S*. *piriei* are less than half the length of the perianth segments, and the flowers are odorless or have a fruity odor (Schmelzer and Gurib-Fakim, 2013).

## 4 Origin and geographic distribution

The genus *Sarcophyte* is native to Africa, and its distribution ranges from Ethiopia to South Africa (Wikimedia Foundation, 2022). *Sarcophyte* comprises a single species with only two plants, whose accepted names are *S*. *sanguinea* and *S*. *piriei*. *Sarcophyte sanguinea* is widespread in eastern and southern Africa, from Ethiopia and Somalia in the north and south to South Africa, while *S*. *piriei* is widespread in Zambia, Malawi, Mozambique, Zimbabwe, and East Africa, from Somalia and Ethiopia to Mozambique and Zimbabwe (Mduduzi and Aluwani, 2017).

**FIGURE 1:**
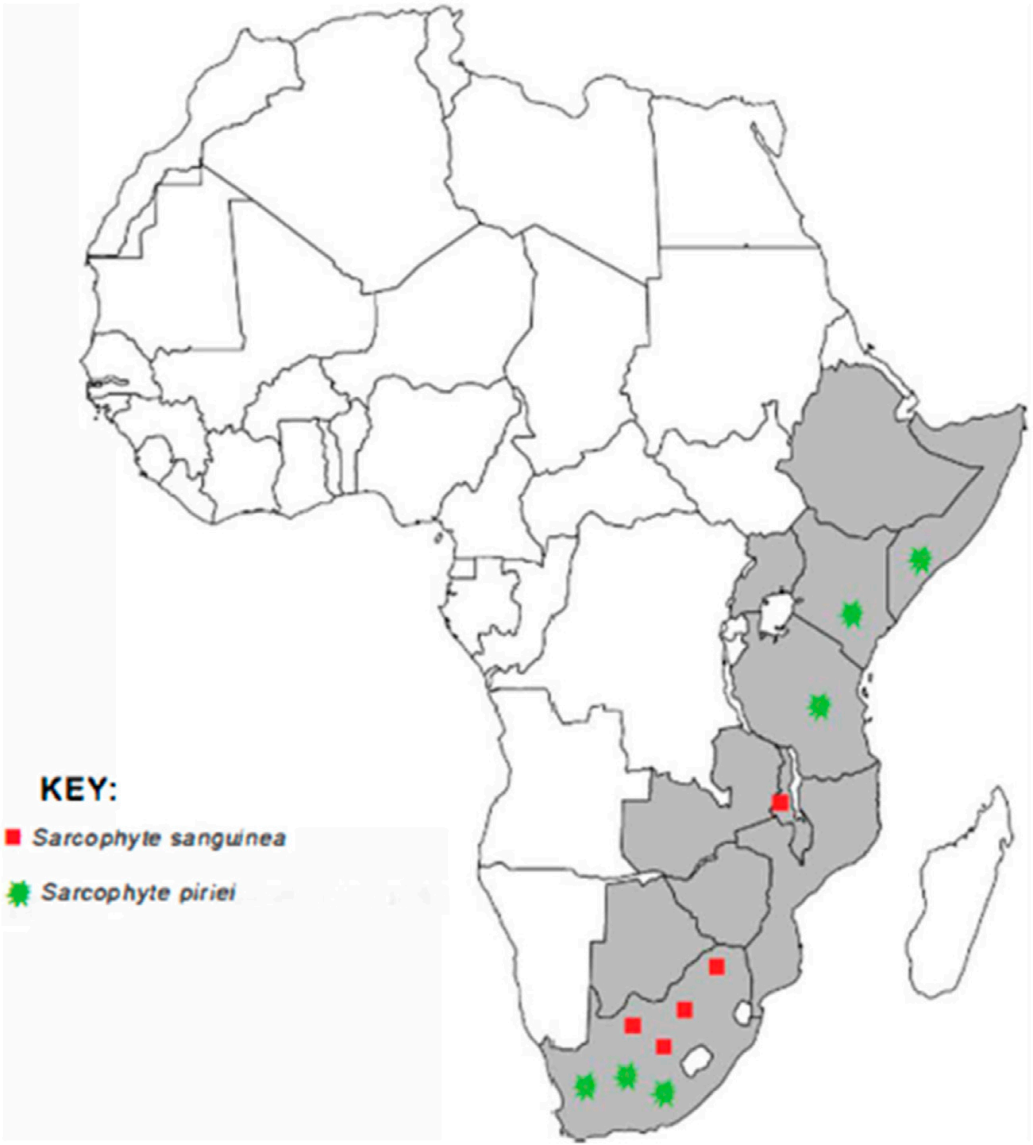
A map of Africa showing the distribution of the two plants of the genus *Sarcophyte* (source: authors).

*Sarcophyte piriei* has been encountered in several parts of Kenya, including Ruwenzori (Kibwezi) (JSTOR, 2022b), Nairobi National Park, Kajiado, Mau Highlands, Tinderet Highlands (Luke, 2017), and Mbeere South (Muriuki, 2011; Onyancha, 2021). Figure 1 shows the distribution of the genus *Sarcophyte* in Africa.

## 5 Ethnomedicinal uses

This review observed that *S*. *sanguinea* is used ethnomedicinally to treat cervical cancer, pimples, dysentery, amenorrhea, swollen glands, gonorrhea, HIV/AIDS, genital warts, sores, shingles, hypertension, and vomiting in humans in various African ethnic communities (Table 1). In addition, it is used to treat black quarter disease and to control lice infestations in pigs (Table 1).

**TABLE 1:** Traditional medicinal uses of and preparation of plants of the genus *Sarcophyte*.

Species	Local name	Part of plant	Disease category	Ethnomedicinal use	Country	Preparation	References
*S*. *sanguinea*	*Mtumbu*	Bulb	Cancer	Cervical cancer	Malawi	Decoction	Ndawonde et al. (2007), Ndawonde et al. (2012)
	*uMavumbuka*	Root	Skin, gastrointestinal, and urogenital diseases	Pimples, dysentery, amenorrhea, diarrhea, and swollen glands	South Africa	Decoction	Ndawonde et al. (2007)
	*uMavumbuka*	Root	Social	Luck	South Africa	Decoction	Ndawonde et al. (2012)
	*uMavumbuka*	Whole plant	Cancer	Not specified	South Africa	Decoction	Sagbo and Mbeng (2018), Sagbo and Otang-Mbeng (2021)
	*uMavumbuka*	Root	Gastrointestinal	Diarrhea	South Africa	Decoction alone or combined with other botanical drugs	De Wet et al. (2010)
	*uMavumbuka*	Whole plant	Urogenital	HIV/AIDS-related infections and gonorrhea, genital warts, and sores	South Africa	Decoction and combination with other botanical drugs	De Wet et al. (2012)
	*uMavumbuka*	Whole plant	Livestock- bacterial disease	Black quarter disease	South Africa	Decoction	Stark et al. (2013)
	*uMavumbuka*	Whole plant	Gastrointestinal	Dysentery and diarrhea	South Africa	Infusion and Decoction	Olajuyigbe (2012)
	*uMavumbuka*	Whole plant	Skin	Shingles and sores	South Africa	Decoction combination with other botanical drugs	De Wet et al. (2016), Britannica (2017), Ramulondi (2017), Odukoya et al. (2022)
	*uMavumbuka*	Whole plant	Metabolic	Hypertension	South Africa	Infusion and decoction	De Wet et al. (2016), Balogun and Ashafa (2019)
	*uMavumbuka*	Root	Ectoparasites, Gastrointestinal	Control of lice infestation in pigs, stomach complaints, and vomiting	South Africa	Not indicated	Nzue (2009)
	*Umavumbuka*	Fruit	Skin	Acne and skin bruises	South Africa	Paste	Dold et al. (2005)
*S*. *piriei*	*Umavumbuka*	Whole plant	Skin	Acne and skin sores and eruptions, burns, wounds, and shingles	South Africa	Decoction in combination with other botanical drugs	De Wet et al. (2013), Maroyi (2017)
	*Ibatikanthi*	Tuber	Cancer	Cancer	Kenya	Decoction	Onyancha (2021)
	*Kimpa cha mwerera*	Tuber	Cancer	Cancer	Tanzania	Decoction	Massimo (2003)
	*Ibatikanthi*	Tuber	Skin	Snakebite antidote	Kenya	Decoction	Muriuki (2011), Omara et al. (2021)
	*Diinsi*	Tuber	Skin, Gastrointestinal	Sore throat, diarrhea, abdominal and menstrual cramps, skin bruises, and toothache	Somalia	Decoction	Schmelzer and Gurib-Fakim (2013)

In addition, *S. piriei* is used to treat cancer, acne, skin sores, skin eruptions, burns, wounds, shingles, snakebites, sore throat, diarrhea, abdominal pain, menstrual pain, skin bruises, and toothache in traditional medicine (Table 1). The extensive application of these plants underscores their perceived therapeutic versatility in traditional medicine. However, the lack of quantitative data or prevalence rates for each application diminishes the accuracy of these claims.

This study also revealed that *S*. *sanguinea* has many diverse applications in South Africa, but only one use was reported in Malawi (Table 1). This regional divergence raises intriguing questions about the cultural, ecological, or historical factors influencing the use of the plant. Conversely, two uses were documented for *S. piriei* in Kenya, and only one was documented in South Africa, Somalia, and Tanzania (Table 1). The observed discrepancies in use reports necessitate a deeper exploration of the factors that contribute to these variations, such as cultural practices, ecological contexts, or methodological differences in ethnomedicinal and ethnobotanical data collection.

## 6 Phytochemistry of *Sarcophyte* sparrm

### 6.1 Identified compounds

**FIGURE 2:**
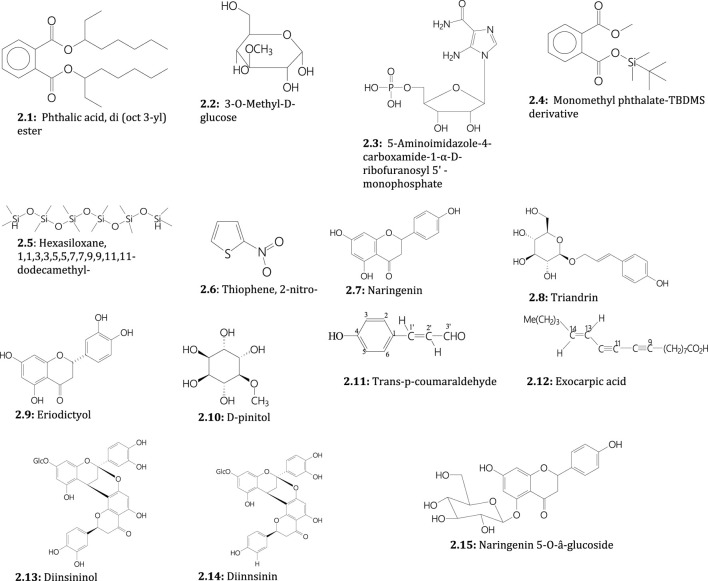
Secondary metabolites isolated from *S*. *piriei* (2.1–2.6), *S. sanguinea* (2.7–2.12), and both plants (2.11–2.15)

Plants of the genus *Sarcophyte* contain various secondary metabolites responsible for various biological activities. Qualitative phytochemical screening of the ethanol and methanol extracts of *S*. *piriei* indicated the presence of alkaloids, flavonoids, tannins, phenolics, saponins, and terpenoids (Mahammed et al., 2020). In addition, quantitative phytochemical analysis of *S. piriei* using gas chromatography-mass spectrometry (GC-MS) revealed the presence of various phytochemicals, including phthalic acid, di (October 3-yl) ester (Figure 2), 3-O-methyl-D-glucose (Figure 2), 5-aminoimidazole-4-carboxamide-1-α-D-ribofuranosyl 5' -monophosphate (Figure 2), monomethyl phthalate (Figure 2), hexasiloxane, 1,1,3,3,5,5,7,7,9,9,11,11-dodecamethyl-(Figure 2), and thiophene, 2-nitro-(Figure 2) as the main compounds (Mbakazi et al., 2022).

On the other hand, narigenin (flavonoid) (Figure 2), triandrin (a phenylpropanoid; a 1-*O*-β-D-glucopyranoside of *p*-coumaryl alcohol) (Figure 2), eriodictyol (flavonone) (Figure 2), D-pinitol (cyclohexanol) (Figure 2), trans-p-coumaraldehyde (cinnamaldehyde) (Figure 2), and exocarpic acid (polyacetylenic fatty acid) (Figure 2) have been isolated from *S*. *sanguinea* (Ogundaini et al., 1996; Schmelzer and Gurib-Fakim, 2013). Notably, three flavonoid glycosides, diinsininol (5,7,3′,4′-tetrahydroxyflavanyl-7-O-â-glucosyl-(4â8:2â-O-7)-eriodictyol) (Figure 2), diinsinin (5,7,3′,4′-tetrahydroxyflavan-7-O-â-glucosyl-(4â8:2â-O-7)-naringenin) (Figure 2) and naringenin 5-O-â-glucoside (Figure 2) have been isolated from tubers/rhizomes of both *S*. *sanguinea* and *S. piriei* and identified using spectroscopic (NMR and U.V.) and spectrometric (FABMS) methods (Ogundaini et al., 1996; Schmelzer and Gurib-Fakim, 2013).

Previous studies have indicated that the flavonoids and fatty acids, among other isolated compounds from the studied plants, possess antioxidant, anticancer, and anti-inflammatory properties (Ríos and Recio, 2005; Adamczak et al., 2020; Moriasi et al., 2021a; Jaradat et al., 2022; Jongrungraungchok et al., 2023; Muruthi et al., 2023).

The identification of these secondary metabolites in *S. piriei* contributes valuable insights into the chemical composition of these plants and offers a foundation for further empirical exploration of their efficacy. Furthermore, the qualitative and quantitative analyses, along with the isolation and identification of specific compounds from these plants, provide a solid basis for future studies exploring the potential pharmacological and therapeutic applications of these secondary metabolites.

### 6.2 Chemical tests

The *in vitro* antioxidant activities of the aqueous and organic extracts of *S. piriei* were determined using various biochemical assay methods. Available data show that these extracts are potent scavengers of the 2,2-diphenyl-1-picrylhydrazyl (DPPH) radical with IC_50_ of 4.26 ± 0.22 μg/mL (methanol extract) and 4.62 ± 0.14 μg/mL (dichloromethane extract) compared with ascorbic acid (IC_50_ = 1.09 ± 0.01 μg/mL) and butylated hydroxytoluene (BHT) (IC_50_ = 3.96 μg/mL) (Mbakazi et al., 2022).

In the 2 -2′-Azino-di-[3-ethylbenzthiazoline sulfonate (ABTS) assay, the methanolic and dichloromethane extracts of *S. piriei* had IC_50_ values of 15.52 ± 0.12 μg/mL and 5.62 ± 0.05 μg/mL, while ascorbic acid and BHT had IC_50_ values of 1.71 ± 0.00 μg/mL and 3.61 ± 0.08 μg/mL, respectively (Mbakazi et al., 2022). In addition, these extracts demonstrated considerable iron chelating effects with IC_50_ values of 1.82 ± 0.01 μg/mL and 3.50 ± 0.09 μg/mL for the methanolic and dichloromethane extracts, respectively, against the standards (ascorbic acid = 0.096 ± 0.04 μg/mL; BHT = 1.00 ± 0.01 μg/mL) (Mbakazi et al., 2022). Furthermore, comparable nitric oxide scavenging activity was observed between the methanolic (IC_50_ = 9.97 ± 0.88 μg/mL) and dichloromethane (IC_50_ = 9.09 ± 0.11 μg/mL) extracts of this plant; however, ascorbic acid (IC_50_ = 2.32 ± 0.01 μg/mL) and BHT (3.22 ± 0.04 μg/mL) were more effective in scavenging the nitric oxide *in vitro* (Mbakazi et al., 2022).

The findings reviewed herein valorize *S. piriei* as a prospective reservoir of antioxidants, exhibiting a spectrum of mechanisms for antioxidant activity (Moriasi et al., 2020b). A comprehensive scientific approach encompassing pharmacokinetic analyses, bioavailability assessments, and toxicity profile investigations is imperative to facilitate the translation of these findings into clinically applicable therapeutic interventions (Wanjiru et al., 2022; Maina et al., 2023).

Moreover, in pursuing therapeutic applications, it is paramount to establish standardized protocols for the extraction processes and dosage formulations. This standardization will ensure consistency and promote the reproducibility of results, thereby increasing the reliability of therapeutic formulations derived from antioxidant-rich extracts. The findings may also help support their utilization as pharmaceuticals or nutraceuticals, a promising avenue for alleviating conditions associated with oxidative stress and fostering general health (Moriasi et al., 2021a).

## 7 Pharmacological activities

The extracts and some of the isolated secondary metabolites from plants of the genus *Sarcophyte* possess biological activities, mainly *in vitro* antimicrobial and cytotoxic activities and *in vivo* anti-inflammatory activity (the only *in vivo* assay reported so far).

### 7.1 Antimicrobial activities

Recently, Mbakazi et al. (Mbakazi et al., 2022), Mohammed et al. (Mahammed et al., 2020), and Dennis et al. (Dennis et al., 2023) reported that the methanol stem extract of *S. piriei* possessed notable *in vitro* antibacterial activities against *Enterococcus avium* (MIC: 0.16 mg/mL; MBC: 1.25 mg/mL), *Staphylococcus aureus, Klebsiella pneumonia,* and *Vibrio fluvialis* (MIC: 0.625 mg/mL; MBC: 2.5 mg/mL) based on disk diffusion and serial broth dilution methods. Also, using the same methods, they reported low activity of the methanol stem extract of *S. piriei*, which was not active against *Escherichia coli* (MIC: 0.625 mg/mL; MBC: >5 mg/mL) in comparison with the standard drug (streptomycin), whose average MIC was 2.2 × 10^−4^ mg/mL (Mahammed et al., 2020; Mbakazi et al., 2022).

Previous studies show that plant extracts with high MIC values (>0.625 mg/mL) are ineffective in eradicating microbes (Mwitari et al., 2013; Clinical Laboratory Standards Institute CLSI, 2022). Therefore, this review reveals that the methanol stem extract of *S. piriei* is more effective against *E. avium* compared to the other microbial strains studied so far (Mwitari et al., 2013; Clinical Laboratory Standards Institute CLSI, 2022). Thus, there is a need to standardize the extracts before subjecting them to bioassays to demystify their efficacy: this will facilitate further antimicrobial investigations and lead to the isolation and optimization of lead molecules for drug development (Gakuya et al., 2020). However, *Serratia marcescens* has been reported to be resistant to the methanol and dichloromethane stem extracts of *S. piriei*, making them of no clinical significance (Mahammed et al., 2020; Mbakazi et al., 2022)

The *in vitro* evaluation of the methanolic stem tuber extract of *S. piriei* using the lactate dehydrogenase (LDH) release assay demonstrated putative membrane-damage-induced cytotoxicity effects against *Listeria ivanovii*, *E. avium,* and *E. coli,* as indicated by the percentage of lactate dehydrogenase release (>50%) (Mbakazi et al., 2022).

Aqueous and methanol-dichloromethane stem extracts of *S*. *sanguinea* demonstrated lower antibacterial activities against *Ureaplasma urealyticum*, *Oligella ureolytica*, *Gardnerella vaginalis*, *Trichomonas vaginalis,* and *Neisseria gonorrhoeae* (MIC: 2.69 mg/mL), indicating negligible antimicrobial efficacy (Naidoo et al., 2013) compared to the standard drug used (ciprofloxacin), whose mean MIC was lower (0.276 μg/mL) (Naidoo et al., 2013) based on microtiter plate dilution method. In addition, the plant extracts studied were inactive against *Candida albicans* (MIC>16 mg/mL), implying a lack of antifungal efficacy compared with amphotericin B (the reference standard), whose MIC was 2.5 μg/mL (Naidoo et al., 2013). Furthermore, an Ames test indicated that the methanol: dichloromethane (1:1) extract of *S. sanguinea* is mutagenic, while the aqueous extract is not mutagenic to the TA98 and TA100 strains of *Salmonella typhimurium* (1,000 and 200 colonies, respectively) when compared with the standard agent (sodium azide) tested on 500 colonies (Ramulondi, 2017).

Considering the variation in antimicrobial efficacy of extracts derived from these plants reported in the reviewed preliminary *in vitro* studies, there is a need to utilize comprehensive and standardized extraction and assay procedures to increase the reliability and reproducibility of the results (Pandey et al., 2014; Altemimi et al., 2017). In addition, these steps will provide a solid foundation for subsequent stages of drug development by systematically identifying lead molecules with therapeutic potential (Maina et al., 2023). Notably, careful attention to various factors, such as the selection of extraction solvents, extraction techniques, and quality control measures, is necessary to establish standardized procedures and optimize the antimicrobial efficacy of plant extracts (Wanjiru et al., 2022).

Furthermore, in-depth research is needed to unravel the specific mechanisms underlying the observed antibacterial and antiprotozoal activities of plant extracts derived from plants of the genus *Sarcophyte*. For instance, exploring the molecular and cellular interactions between bioactive secondary metabolites in target microorganisms will provide valuable insights into their mode of action and provide a solid basis for rational drug design and development (Abreu et al., 2012; Bor et al., 2016). Furthermore, it is crucial to determine the potential synergistic effects between different plant extracts and active secondary metabolites to uncover synergies that boost therapeutic efficacy and their potential applications in clinical practice (Abreu et al., 2012).

### 7.2 Cytotoxicity studies

Various studies utilizing different assay methods indicate variations in the cytotoxic effects of extracts derived from the investigated plants. The aqueous and methanol:dichloromethane (1:1) extracts of *S*. *sanguinea* were previously reported to be non-toxic to the normal human embryonic kidney epithelial cell line (Graham, HEK-293), recording 100% cell viability at a concentration of 100 μg/mL using the 3-(4,5-dimethylthiazol-2-yl)-2,5-diphenyltetrazolium bromide (MTT) method (Naidoo et al., 2013). In addition, the methanol and dichloromethane extracts of *S. piriei* have shown low cytotoxic effects with CC_50_ ˃ 200 μg/mL (normal human embryonic kidney (HEK293) and human breast endocrine cells (SKBR-3) compared to the standard (doxorubicin) (CC_50_ = 1.65 μg/mL) in the MTT assay (Mbakazi et al., 2022).

Elsewhere, extracts of *S*. *sanguinea* have been demonstrated to be non-toxic to brine shrimp larvae (LC_50_ ˃ 2000 μg/mL) compared to potassium dichromate (the positive control agent), which was highly toxic to brine shrimp larvae at a concentration of 2 μg/mL, resulting in 100% mortality (Ramulondi, 2017). *In vitro* toxicity studies for conventional drugs and botanical drug interactions of the aqueous stem extract of *S. sanguinea* have shown to cause a 15% inhibition of *β*-glucuronidase at 10 μg/mL (Ramulondi, 2017), demonstrating its potential efficacy in inhibiting colonic genotoxicity, induced by deconjugation of drug and xenobiotic glucuronides in the gastrointestinal (GI) tract (Awolade et al., 2020). However, the same extract inhibits carboxylesterase by 38% and cytochrome P450 3A4 (CYP3A4) by 82% at a concentration of 10 μg/mL (Ramulondi, 2017), which may negatively impact xenobiotic metabolism in the GIT and liver (Sevrioukova and Poulos, 2013; Singh et al., 2021).

This review noted that the aqueous and dichloromethane:methanol extracts of *S. sanguinea* extracts possess *in vitro* antiproliferative activity against the human hepatocarcinoma cell line (HepG2/C3A), with approximately 69% cell death at a concentration of 100 μg/ml as compared to the positive control (melphalan), which revealed 100% cell death (Ramulondi, 2017). In addition, *S. piriei* extracts demonstrated lower *in vitro* antiproliferative activities against human colorectal carcinoma (Caco-2) and human hepatocellular carcinoma (HepG2) cells (CC_50_: 221 to >250 μg/mL) than the positive control drug (doxorubicin) (CC_50_ = 2.27 μg/mL) (Mahmoud et al., 2011; Mbakazi et al., 2022). However, it is arguable that since various medicinal plants are often combined in traditional medicine to treat cancer, among other devastating conditions, the efficacy of these extracts may be improved when combined (Maina et al., 2023), or when another solvent, especially water, which is commonly used in traditional medicine, is used (Sri Widyawati et al., 2014; Truong et al., 2019).

Based on the reported findings, in-depth *in vivo* studies are necessary to substantiate and validate the observed effects of *S*. *sanguinea* extracts against cancer cells using different standardized experimental designs with appropriate animal models and controlled conditions to elucidate their pharmacokinetic and pharmacodynamic aspects in a physiological context. Research shows that the optimization of extraction methodologies represents a critical avenue for refining the therapeutic potential of plant extracts (Handa et al., 2008; Hamuel, 2012; Altemimi et al., 2017; Abubakar and Haque, 2020), such as those from *S*. *sanguinea* and *S. piriei*. This optimization process should consider different extraction solvents, temperature, duration, and pressure to attain maximum yields of the bioactive secondary metabolites while preserving their stability and bioavailability (Dai and Mumper, 2010; Pandey et al., 2014; Altemimi et al., 2017). The use of advanced techniques, such as chromatographic and spectroscopic analyses, can assist in characterizing and quantifying the extracted secondary metabolites, offering valuable insights into their pharmacological activities (Handa et al., 2008).

Moreover, exploring potential synergies with other medicinal plants may help to enhance the therapeutic efficacy of the investigated plant extracts by improving their pharmacokinetics and spectrum of therapeutic effects, as reported in previous studies (Zhang et al., 2019; Ndung et al., 2018; Karki et al., 2021; Jouda, 2013). Systematic screening of compatible plant combinations, guided by traditional knowledge and modern pharmacological principles, would be critical in identifying synergistic relationships that amplify the therapeutic benefits of the more promising extracts.

In addition, traditional knowledge systems often incorporate specific solvents, and investigating the implications of such choices on the extraction of bioactive secondary metabolites can offer valuable insights into the efficacy of these preparations in treating the conditions claimed (Do et al., 2014). Thus, collaborative efforts involving traditional practitioners and modern scientists can bridge the gap between empirical knowledge and scientific validation, facilitating a holistic understanding of the therapeutic potential inherent in traditional practices.

### 7.3 Anti-inflammatory activity

Recent *in vivo* studies have shown that the aqueous rhizome extract of *S. piriei* significantly inhibited 50% of carrageenan-induced edema in rodents at a dose of 1,000 mg/kg after 10 h (Ogundaini et al., 1996). In addition, this extract effectively inhibited *in vitro* prostaglandin synthesis (IC_50_ = 0.2 mg/mL) comparable to aspirin, suggesting its anti-inflammatory efficacy (Ogundaini et al., 1996). Moreover, diinsininol and diinsinin, isolated from the tuber/rhizome of *S. sanguinea*, inhibit *in vitro* prostaglandin synthesis with IC_50_ values of 9.20 and 13.14 µM, respectively, and platelet-activating factor-induced exocytosis with IC_50_ values of 49 and 39 μM, respectively (Ogundaini et al., 1996; Attiq et al., 2018).

This review highlights the promising anti-inflammatory properties exhibited by the aqueous rhizome extracts of *S*. *sanguinea* and *S. piriei*, which are mainly attributed to diinsininol and diinsinin. While these findings hold potential for therapeutic applications, a successful translation into clinical practice necessitates a comprehensive approach that includes rigorous pre-clinical investigations, an intricate understanding of pharmacokinetics and pharmacodynamics, and robust clinical trials to establish safety and efficacy in human subjects (Shakya, 2016; Jamshidi-Kia et al., 2018).

Extensive pre-clinical studies are essential to elucidate the safety profile and efficacy in various animal models to ensure reproducibility and consistency of the observed anti-inflammatory effects and to provide a solid foundation for subsequent clinical exploration (Nasri and Shirzad, 2013; Moriasi et al., 2021b). In addition, a nuanced understanding of the pharmacokinetic and pharmacodynamic characteristics of diinsininol and diinsinin, including absorption, distribution, metabolism, and excretion within the biological system, is imperative (Raj and Raveendran, 2019). Furthermore, determining the optimal dosage and route of administration and assessing potential side effects and drug interactions are crucial to determining their safety profile and maximizing their therapeutic benefit (Turner et al., 2011; Kathare et al., 2021).

In addition, well-designed clinical trials involving human subjects are indispensable to validating the anti-inflammatory effects observed in pre-clinical studies. These trials should rigorously adhere to ethical standards, involve diverse participant populations to ensure broader applicability, and employ robust methodologies to yield reliable and generalizable results (Knowles, 2014). The amalgamation of these scientific endeavors is integral to the successful translation of the anti-inflammatory properties of the aqueous rhizome extracts of *S*. *sanguinea* and *S. piriei* and their constituent secondary metabolites into clinically effective therapeutic interventions.

## 8 Limitations

Our review, although comprehensive, has some limitations. The few reviewed materials may not adequately provide sufficient information on the local and traditional medicinal uses and the pharmacology and phytochemistry of the explored plants. In addition, we did not independently verify the efficacy reported in the reviewed literature; therefore, we could not offer specific applications in clinical practice. Moreover, we observed that only preliminary studies were performed involving *in vitro* models; therefore, they may not mirror *in vivo* effects, the reports of which are currently unavailable.

## 9 Conclusion and future directions

This study summarizes for the first time the traditional uses, phytochemistry, and pharmacology of plants of the genus *Sarcophyte*. This review noted that extracts of these plants have wide traditional medicinal applications, ranging from the treatment of infectious diseases, disorders of the skin, gastrointestinal and urogenital tracts, and cancer, among others. Also, their extracts possess various *in vitro* cytotoxic effects against normal cell lines, cancer cell lines, brine shrimp nauplii, and some microbes. In addition, the extracts of these plants possess considerable antioxidant and anti-inflammatory activities. In particular, the available literature revealed that only the anti-inflammatory activity of the pure isolated secondary metabolites had been performed, leaving a significant lack of information on other bioactivities, including those of the other phytochemicals, which should be explored empirically.

Based on the current review, we recommend further empirical investigations to evaluate the toxic effects and pharmacological activities of the extracts and isolated secondary metabolites using a bio-assay-guided approach to decipher their potential. In addition, bioactivity-guided fractionation, identification, and characterization of the secondary metabolites, mainly against pathogenic microbes, oxidative stress, inflammation, and cancer, among others, should be conducted based on the documented ethnomedicinal claims.

## Abbreviations

ABTS, 2 -2′-Azino-di- [3-ethylbenzthiazoline sulfonate; BHT, Butylated hydroxytoluene; BSLT, Brine shrimp lethality test; DPPH, 2,2-diphenyl-1-picrylhydrazyl; EIMS, Electron Ionization Mass Spectrometry; FABMS, Fast Atom Bombardment Mass Spectrometry; FABMS, Fast atom bombardment mass spectrometry; IC_50_, Median Inhibitory concentration; IR, Infra-red; LC_50_, Median Lethal Concentration; NMR, Nuclear magnetic resonance; PAF, Platelet-activating factor; TBDMS, *tert*-Butyldimethylsilyl; UV, Ultraviolet.
